# Genetic diversity and characteristics of high-level tigecycline resistance Tet(X) in *Acinetobacter* species

**DOI:** 10.1186/s13073-020-00807-5

**Published:** 2020-12-07

**Authors:** Chong Chen, Chao-Yue Cui, Jun-Jun Yu, Qian He, Xiao-Ting Wu, Yu-Zhang He, Ze-Hua Cui, Cang Li, Qiu-Lin Jia, Xiang-Guang Shen, Ruan-Yang Sun, Xi-Ran Wang, Min-Ge Wang, Tian Tang, Yan Zhang, Xiao-Ping Liao, Barry N. Kreiswirth, Shi-Dan Zhou, Bin Huang, Hong Du, Jian Sun, Liang Chen, Ya-Hong Liu

**Affiliations:** 1 National Risk Assessment Laboratory for Antimicrobial Resistance of Animal Original Bacteria, College of Veterinary Medicine, South China Agricultural University, Guangzhou, China; 2 Guangdong Laboratory for Lingnan Modern Agriculture, South China Agricultural University, Guangzhou, China; 3 Guangdong Provincial Key Laboratory of Veterinary Pharmaceutics Development and Safety Evaluation, South China Agricultural University, Guangzhou, China; 4 Guangdong Enterprise Key Laboratory for Animal Health and Environmental Control, WENS Foodstuff Group Co Ltd, Xinxing, China; 5 Center for Discovery and Innovation, Hackensack Meridian Health, Nutley, NJ USA; 6 Intensive Care Unit, Huizhou Municipal Central Hospital, Huizhou, China; 7 Department of Laboratory Medicine, The First Affiliated Hospital of Sun Yat-sen University, Guangzhou, China; 8 Department of Clinical Laboratory, The Second Affiliated Hospital of Soochow University, Suzhou, China; 9 Hackensack Meridian School of Medicine at Seton Hall University, Nutley, NJ USA

**Keywords:** *tet*(X), *bla*_NDM-1_, Tigecycline resistance, IS*CR2*, *Acinetobacter* species, Flavobacteriaceae bacteria, Ecological niches

## Abstract

**Background:**

The recent emergence and dissemination of high-level mobile tigecycline resistance Tet(X) challenge the clinical effectiveness of tigecycline, one of the last-resort therapeutic options for complicated infections caused by multidrug-resistant Gram-negative and Gram-positive pathogens. Although *tet*(X) has been found in various bacterial species, less is known about phylogeographic distribution and phenotypic variance of different genetic variants.

**Methods:**

Herein, we conducted a multiregional whole-genome sequencing study of *tet*(X)-positive *Acinetobacter* isolates from human, animal, and their surrounding environmental sources in China. The molecular and enzymatic features of *tet*(X) variants were characterized by clonal expression, microbial degradation, reverse transcription, and gene transfer experiments, while the *tet*(X) genetic diversity and molecular evolution were explored by comparative genomic and Bayesian evolutionary analyses.

**Results:**

We identified 193 *tet*(X)-positive isolates from 3846 samples, with the prevalence ranging from 2.3 to 25.3% in nine provinces in China. The *tet*(X) was broadly distributed in 12 *Acinetobacter* species, including six novel species firstly described here. Besides *tet*(X3) (*n* = 188) and *tet*(X4) (*n* = 5), two *tet*(X5) variants, *tet*(X5.2) (*n* = 36) and *tet*(X5.3) (*n* = 4), were also found together with *tet*(X3) or *tet*(X4) but without additive effects on tetracyclines. These *tet*(X)-positive *Acinetobacter* spp. isolates exhibited 100% resistance rates to tigecycline and tetracycline, as well as high minimum inhibitory concentrations to eravacycline (2–8 μg/mL) and omadacycline (8–16 μg/mL). Genetic analysis revealed that different *tet*(X) variants shared an analogous IS*CR2*-mediated transposon structure. The molecular evolutionary analysis indicated that Tet(X) variants likely shared the same common ancestor with the chromosomal monooxygenases that are found in environmental Flavobacteriaceae bacteria, but sequence divergence suggested separation ~ 9900 years ago (7887 BC), presumably associated with the mobilization of *tet*(X)-like genes through horizontal transfer.

**Conclusions:**

Four *tet*(X) variants were identified in this study, and they were widely distributed in multiple *Acinetobacter* spp. strains from various ecological niches across China. Our research also highlighted the crucial role of IS*CR2* in mobilizing *tet*(X)-like genes between different *Acinetobacter* species and explored the evolutionary history of Tet(X)-like monooxygenases. Further studies are needed to evaluate the clinical impact of these mobile tigecycline resistance genes.

**Supplementary information:**

The online version contains supplementary material available at 10.1186/s13073-020-00807-5.

## Background

Tetracycline antibiotics have been extensively used in prophylaxis and treatment of human and animal infections, as well as at subtherapeutic levels as growth-promoters in animal feed [1, 2]. The third-generation tetracycline antibiotic, tigecycline, is regarded as one of the last-resort antibiotics to treat clinical multidrug-resistant (MDR) bacterial infections. It exhibits an expanded spectrum of activities against both Gram-negative and Gram-positive bacteria, including carbapenem-resistant Enterobacteriaceae (CRE) and *Acinetobacter baumannii* (CRAB), methicillin-resistant *Staphylococcus aureus* (MRSA), and vancomycin-resistant *Enterococcus* (VRE) strains [3, 4], and is classified as a critically important antimicrobial by the World Health Organization.

However, the recent emergence of plasmid-mediated high-level tigecycline resistance mechanisms, Tet(X3), Tet(X4), and Tet(X5), raises the concern that this last-resort antibiotic may be ineffective, further limiting clinical treatment choices [5–7]. Tet(X), a flavin-dependent monooxygenase, is capable of degrading all tetracycline antibiotics by hydroxylation, representing a unique enzymatic tetracycline inactivation mechanism [8, 9]. Thus far, *tet*(X) genes [especially *tet*(X3)–*tet*(X5)] have been detected in over 16 different bacterial species from various ecological niches of humans, migratory birds, food-producing animals, and their neighboring environments, with *Acinetobacter* spp. and *Escherichia coli* the most predominate species [5, 7, 10, 11]. Nevertheless, the original source and evolutionary history of *tet*(X) genes remain poorly understood. In addition, our previous surveillance study on *E. coli* revealed a low prevalence of *tet*(X)-positive *E. coli* isolates from various sources in China, with *tet*(X4) the only variant detected [6]. Conversely, it remains unclear how much *Acinetobacter* spp. strains contribute to the dissemination of *tet*(X).

The genus *Acinetobacter* is a heterogeneous group and comprised of more than 60 bacterial species [12], which are ubiquitous in the nature but can also cause serious infections in hospital settings [13, 14]. Extensively drug-resistant (XDR) *Acinetobacter* species, especially CRAB, have emerged as a clinically troublesome pathogen [15]. In this study, we investigated the prevalence of *tet*(X) genes in *Acinetobacter* spp. isolates from human, migratory bird, pig, and surrounding environmental samples in China, and explored the genetic diversity and characteristics of *tet*(X) genes, plasmids, and strains.

## Methods

### Sample collection and bacterial isolation

We conducted a multiregional study to investigate the prevalence of *tet*(X) genes in *Acinetobacter* isolates from human, pig, migratory bird, and surrounding environmental samples between May 2015 and May 2018. There is no self-selection bias that may be present during the sample collection. Briefly, the animal stool samples were randomly collected, with approximate 50 samples per pig farm (*n* = 2083) or 150 samples per migratory bird habitat (*n* = 863). If possible, the soil (*n* = 182), dust (*n* = 170), sewage (*n* = 136), water (*n* = 54), and vegetable (*n* = 59) samples were also collected at least three per site. In addition, the human specimens (urine, *n* = 175; nasal swabs, *n* = 64; rectal swabs, *n* = 60) were collected from Guangdong province for clinical investigation. Subsequently, the feces of pigs and migratory birds, soils, dusts, and chopped vegetables were suspended in sterile saline at a weight/volume ratio of 1:5, respectively. Meanwhile, the nasal and rectal swabs of inpatients were directly discharged into 1.5 mL of sterile saline. For the treated samples, together with sewage, water, and urine of physical examination people, 100 μL of them was used for the next bacterial isolation.

A total of 3846 none-duplicate samples were collected from one tertiary-care hospital (*n* = 299), five migratory bird habitats (*n* = 972), and 33 intensive pig farms (*n* = 2575) in 14 provinces and municipalities in China. These samples were then selected by CHROMagar™ *Acinetobacter* plates (CHROMagar, France) containing tigecycline (2 μg/mL), and the isolates were screened for *tet*(X3) and *tet*(X4) by PCR amplification with primers listed in the supplementary information (Additional file 1: Table S1). In addition, a random collection of 402 *A. baumannii* clinical isolates from two hospitals in Guangdong and Jiangsu provinces was also screened as described above. All these *tet*(X)-positive *Acinetobacter* spp. strains were further characterized by whole-genome sequencing (described as below).

### Antimicrobial susceptibility testing

Minimum inhibitory concentrations (MICs) of ten antimicrobials for *tet*(X)-positive *Acinetobacter* spp. isolates were determined by twofold agar dilution method and interpreted according to the Clinical and Laboratory Standards Institute (CLSI) guideline [16]. In brief, the antibiotic to be tested was diluted by 1 mL of sterile ddH_2_O to make a series of concentrations, followed by mixing with 19 mL of fresh Mueller-Hinton (MH) agar to produce plates in which the final antibiotic concentrations represented a 2-fold dilution series. The bacterial suspension (~ 10^5^ cfu) was then spotted on MH plates and incubated at 35 °C for 20 h. The lowest concentration of antibiotics that prevented bacterial growth was considered to be the MIC. Additionally, MICs of tigecycline, eravacycline, and omadacycline were determined by broth microdilution method. In particular, the breakpoint of tigecycline was interpreted according to the United States Food and Drug Administration (USFDA) criteria for Enterobacteriaceae bacteria as previously reported [17]. *E. coli* strain ATCC 25922 was used as the quality control strain.

### Whole-genome sequencing (WGS) and bioinformatics analysis

The genomic DNA of 193 *tet*(X)-positive *Acinetobacter* spp. strains was extracted and sequenced using the Illumina HiSeq platform (Novogene, China). The raw sequence data were then assembled by SPAdes version 3.12.0 [18]. To obtain complete sequences, five representative *Acinetobacter* spp. strains, namely *Acinetobacter* Clade_U6 10FS3-1 [*tet*(X3)-positive], *Acinetobacter* Clade_U1 YH12138 [*tet*(X3)- and *tet*(X5.2)-positive], *A. piscicola* YH12207 [*tet*(X3)- and *tet*(X5.3)-positive], *A. indicus* Q186-3 [*tet*(X4)- and *tet*(X5.2)-positive], and *A. indicus* C15 [*tet*(X3)- and *bla*_NDM-1_-positive], were further subjected to Oxford Nanopore sequencing (Nextomics, China), followed by assembling with Unicycler version 0.4.8 [19]. To determine the bacterial species, pair-wised average nucleotide identities (ANIs) were calculated using FastANI and Mash [20, 21], and compared with *Acinetobacter* genomes from the National Center for Biotechnology Information (NCBI) RefSeq database [22]. A cutoff of > 95% and < 83% ANI values was used to determine intra-species and inter-species boundaries, respectively [20]. A Mash distance tree was generated using mashtree [23].

Gene prediction and annotation were performed according to the NCBI Prokaryotic Genome Annotation Pipeline [24]. The heatmap of antibiotic resistance genes was created using the R package pheatmap version 1.0.12 [25]. The visual representation of *tet*(X)-carrying plasmids was generated with DNAPlotter version 1.11 [26]. A further BLASTn/BLASTp analysis of *tet*(X5.3) against the NCBI database identified over 200 homologous sequences with high-scoring hits (*E* value < 2e^−65^) [27]. Subsequently, the amino acid sequences of 54 non-duplicated Tet(X) proteins and monooxygenases were used to estimate the substitution rate among 404 sites and produce a divergence time tree by BEAST version 2.6.0, with tip-dates defined as the years of isolation or submission [28]. In brief, we analyzed the data under a Bayesian framework using the strict clock+Gamma site or relaxed clock+Gamma site model, and the latter model was a better one because it had higher effective sample size values (ESS, > 200). To test whether population growth rates differed between different lineages, a Markov Chain Monte Carlo method was used for posterior probability distributions. Three independent runs, with chain lengths 100,000,000 and 20% burn-in, were conducted to confirm the convergence of Bayesian time-calibrated phylogenetic analyses. Parameter convergence was visualized by Tracer version 1.7 [29]. The collected trees were then annotated into a maximum clade credibility tree using TreeAnnotator version 2.6.0 in the BEAST package.

### Cloning experiments

Firstly, the original *tet*(X5) (accession number: CP040912) and putative ancestral flavin-dependent monooxygenase gene *fmo* (CP014021) were artificially synthesized according to the reference sequences from NCBI. The *tet*(X3), *tet*(X4), *tet*(X5.2), and *tet*(X5.3) variants were PCR amplified from isolates Clade_U6 10FS3-1, *A. indicus* Q278-1, Clade_U1 YH12138, and *A. piscicola* YH12207, respectively. These genes were then cloned into the plasmid vector pBAD24 under an arabinose inducible P_BAD_ promoter as our previous description [6]. In addition, the recombinant plasmids co-harboring *tet*(X3)/*tet*(X5.2) or *tet*(X3)/*tet*(X5.3) were further constructed, using pBAD24+*tet*(X3) as the plasmid skeleton. All these constructs were selected on Luria-Bertani (LB) agar plates containing 100 μg/mL ampicillin (resistance encoded by *amp* on the pBAD24 backbone), and confirmed by PCR detection and Sanger sequencing for *tet*(X) genes (Additional file 1: Table S1). Susceptibility testing for six tetracyclines (namely tetracycline, doxycycline, minocycline, tigecycline, eravacycline, and omadacycline) was performed by broth microdilution with the addition of 0.1% l-arabinose.

### Eravacycline degradation assay

The eravacycline degradation ability of five *tet*(X) variant constructs and their parental strains was initially determined by agar well diffusion assay in triplicate [6]. In brief, the MH agar plate surface was inoculated by spreading 100 μL of overnight culture of eravacycline-susceptible *Bacillus stearothermophilus* ATCC 7953 (0.5 McFarland), and the well with a diameter of 6 to 8 mm was punched aseptically with a sterile cork borer. The *tet*(X) constructs were cultured in 5 μg/mL eravacycline and 0.1% l-arabinose at 37 °C for 8 h. Supernatant (20 μL) was then transferred into the agar well and incubated at 60 °C for 16 h. The media only containing eravacycline and the samples treated with *E. coli* JM109 carrying an empty plasmid pBAD24 were used as the control groups. The parental strains were examined similarly, but without the addition of 0.1% l-arabinose during incubation.

In addition, the degradation levels of eravacycline by *tet*(X5.2) and *tet*(X5.3) were also quantified by liquid chromatography-tandem mass spectrometry (LC-MS/MS) in quadruplicate as previously described [6]. Briefly, the *tet*(X) clones were incubated in 4 mL of optimized M9 media with eravacycline (2 μg/mL) and l-arabinose (0.1%) at 200 rpm for 16 h. Following centrifugation at 10,000 rpm for 2 min, the supernatant was then passed through a 0.22-μm filter and subjected to LC-MS/MS quantification. Meanwhile, the previously reported *tet*(X3), *tet*(X4), and *tet*(X5) genes served as the positive controls for comparative analyses, while *E. coli* JM109 carrying pBAD24 was used as a negative control. The statistical analysis was conducted using an unpaired, two-sided *t* test. The linear range of the standard curve for eravacycline was from 10 to 500 ppb, with *r*^2^ = 0.994.

### Quantitative reverse transcription-PCR (qRT-PCR)

The transcript expression levels of different *tet*(X) variants in a tandem structure were determined by qRT-PCR in triplicate. Total bacterial RNA of *Acinetobacter* Clade_U1 YH12138 and *A. piscicola* YH12207 was obtained using the E.Z.N.A. Bacterial RNA Kit (OMEGA, China), respectively, and then reversely transcribed into cDNA using the M-MLV First Strand cDNA Synthesis Kit (OMEGA, China). Quantitative real-time PCR was performed with SYBR Premix Ex Taq (Takara, China) on a LightCycler 96 instrument (Roche, Switzerland) as previously reported [30]. 16S rRNA was used as the endogenous control (Additional file 1: Table S1). Relative expression levels between *tet*(X3) and *tet*(X5.2) or *tet*(X5.3) were calculated using the 2^-ΔΔCT^ method [31].

### Mobilization of *tet*(X)-mediated tigecycline resistance

The transferability of tigecycline resistance mediated by *tet*(X3), *tet*(X4), *tet*(X5.2), and *tet*(X5.3) was determined by filter mating using representative *Acinetobacter* spp. isolates as the donor strains and *A. baylyi* ADP1 (rifampicin-resistant), *E. coli* C600 (streptomycin-resistant), and clinical *E. coli* 1314 (meropenem-resistant) strains as the recipient strains. The transconjugants were selected on LB agar plates containing tigecycline (2 μg/mL) in combination with rifampin (100 μg/mL), streptomycin (1500 μg/mL), or meropenem (2 μg/mL), respectively.

To confirm the IS*CR2*-mediated *tet*(X) transfer, a transposition assay of *tet*(X) was conducted as previously reported for *mcr-1* [32]. The putative transposition unit of *tet*(X3) in Clade_U6 10FS3-1, namely Δ*tpnF*-*tet*(X3)-hp-hp-IS*CR2*, was cloned in a suicide plasmid pSV03 using the ClonExpress Ultra One Step Cloning Kit (Vazyme, China) (Additional file 1: Table S1) and then transformed into competent *E. coli* WM3064. Subsequently, the transformant was treated by filter mating with the recipient *E. coli* K-12 strain MG1655 (*recA*∷Km) and *A. baylyi* ADP1, and the transposon-insertion mutants were selected by LB agar plates containing tigecycline (2 μg/mL) as well as kanamycin (30 μg/mL) or rifampin (100 μg/mL).

All the putative transconjugants and transposon mutant strains were screened for *tet*(X) genes by PCR and Sanger sequencing (Additional file 1: Table S1). The recipient strain backgrounds were confirmed by PCR-fingerprints for *A. baylyi* and enterobacterial repetitive intergenic consensus PCR (ERIC-PCR) for *E. coli* [33, 34]. Transfer efficiency was calculated based on colony counts of the transconjugant, transposon mutant, and recipient cells in triplicate [35].

## Results

### Prevalence of *tet*(X)-positive *Acinetobacter* spp. strains in China

**Fig. 1:**
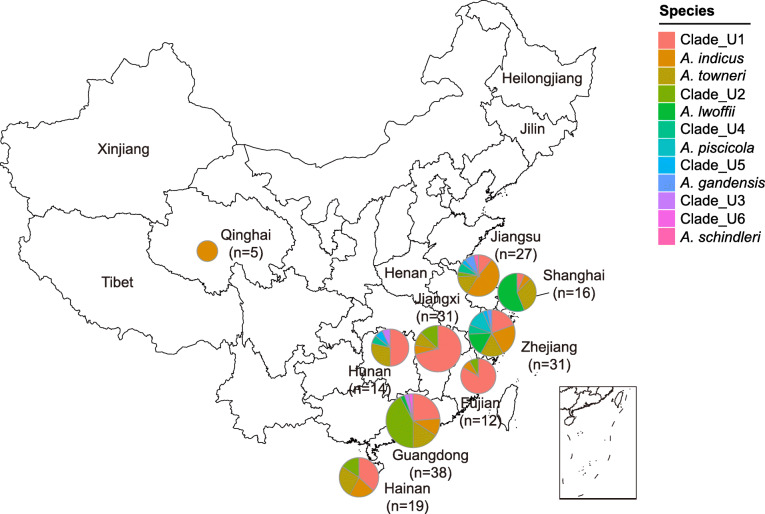
Distribution of *tet*(X)-positive *Acinetobacter* spp. strains in China. The 14 sampling provinces and municipalities are marked. The bacterial number per region and corresponding proportion are also indicated

In this study, a total of 3846 samples were collected from 14 provinces and municipalities in China from pig farms, migratory bird habitats, and human specimens. PCR assays identified a total of 193 *tet*(X3)- or *tet*(X4)-positive *Acinetobacter* spp. isolates (5.0%, 193/3846) from pig farms (7.3%, 187/2575), migratory birds (0.5%, 5/972), and human samples (0.3%; 1/299) in nine provinces and municipalities (Fig. 1; Additional file 1: Table S2). Among them, 188 isolates were positive for *tet*(X3) and five for *tet*(X4). The *tet*(X3) gene was widely distributed in southern and eastern China, including Hainan (25.3%, 19/75), Shanghai (18.8%, 16/85), Jiangsu (13.2%, 27/204), Hunan (12.6%, 14/111), Zhejiang (7.6%, 31/406), Jiangxi (4.7%, 31/666), Guangdong (4.2%, 38/914), and Fujian (3.1%, 12/390), while *tet*(X4) was only found in Qinghai (2.3%, 5/214) (Fig. 1). An *A. lwoffii* strain (YH18001) recovered from a urine sample in a healthy individual in Huizhou, Guangdong, carried *tet*(X3). The screening of 402 *A. baumannii* clinical isolates from two hospitals in Guangdong and Jiangsu provinces did not identify *tet*(X)*-*positive isolates.

**Fig. 2:**
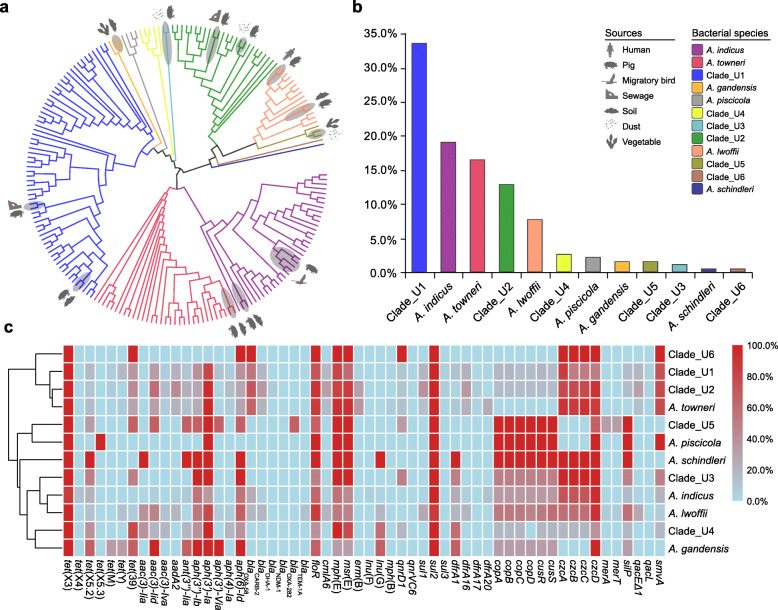
Genomic characteristics of *tet*(X)-positive *Acinetobacter* species. **a** Mash distance relationship of 193 *tet*(X)-positive *Acinetobacter* spp. isolates. The branch lines are colored based on the bacterial species as listed on the right. The bacterial sources of representative isolates are illustrated by silhouettes. The closely related isolates from different sources are highlighted by shading. **b** Distribution of *tet*(X)-positive strains in 12 *Acinetobacter* species. **c** Heatmap of antibiotic resistance genes in 12 *Acinetobacter* species. The color of each box represents the percentage of resistance genes in corresponding species, ranging from 0.0 (cyan) to 100.0% (red)

The 193 *tet*(X)-positive strains were widely distributed in 12 different *Acinetobacter* species, including six putative novel species. An ANI analysis showed that 101 genomes could not be assigned into any known *Acinetobacter* species, but they demonstrated 84–90% FastANI and Mash-based ANI values against known *Acinetobacter* species from the NCBI RefSeq database, suggesting that they likely belong to novel species in the *Acinetobacter* genus. The 101 genomes were clustered in six groups, with > 95% ANI values in each group. In this study, we tentatively named the six novel species as *Acinetobacter* Clade_U1 to Clade_U6 (Fig. 2a). Interestingly, the *Acinetobacter* Clade_U1 was the most predominant *tet*(X)-harboring species (33.7%, 65/193), followed by *A. indicus* (16.6%, 32/193), *A. towneri* (16.6%, 32/193), Clade_U2 (13.0%, 25/193), and *A. lwoffii* (7.8%, 15/193) (Fig. 2b).

It should be noted that these *tet*(X3)-positive strains were detected in various sources in pig farms, including soil (9.4%, 12/127), pig (7.6%, 158/2083), sewage (6.6%, 9/136), vegetable (5.1%, 3/59), and dust (2.9%, 5/170) samples, suggesting *tet*(X3) is widely spread in the intensive pig farms in China (Additional file 1: Table S2). The genome of aforementioned human *A. lwoffii* isolate YH18001 was closely related to some *A. lwoffii* isolates collected from pig stool samples (e.g., YH12105 from a pig in Shanghai) (Fig. 2a). In addition, the *tet*(X4) gene was detected in five *A. indicus* strains from migratory birds [bar-headed goose (*Anser indicus*)] in Qinghai province (Fig. 1; Additional file 1: Table S2). Notably, three out of them were clustered together with some *A. indicus* strains (e.g., YH12090 and YH12091) isolated from pig sources in Guangdong province (Fig. 2a).

### Identification of *tet*(X5.2) and *tet*(X5.3) variants in *Acinetobacter* spp.

Genomic sequencing of *tet*(X3)- or *tet*(X4)-positive *Acinetobacter* spp. strains identified two additional *tet*(X)-like genes. Both of them were 1137 bp open reading frames (ORFs), encoding 378 amino acid proteins (Additional file 1: Figure S1), which showed 95% [designated as Tet(X5.2) herein] and 96% [Tet(X5.3)] amino acid sequence identities to the first reported Tet(X5) (CP040912) in a clinical *A. baumannii* strain, respectively. Homology modeling of Tet(X5), Tet(X5.2), and Tet(X5.3) illustrated an overall analogous architecture that consisted of the substrate-binding domain, FAD-binding domain, and C-terminal α-helix (Additional file 1: Figure S2). Similar to previously reported *tet*(X3), *tet*(X4), and *tet*(X5), the tigecycline MICs of *tet*(X5.2) (8 μg/mL) and *tet*(X5.3) (8 μg/mL) constructs increased 64-fold when compared with that of the *E. coli* JM109 control carrying the empty pBAD24 vector (0.13 μg/mL) (Additional file 1: Table S3). The same tigecycline MIC (16 μg/mL) was observed between *tet*(X3) and *tet*(X3)/*tet*(X5.2) or *tet*(X3)/*tet*(X5.3) constructs. In addition, these constructs exhibited at least 64-fold increases of MICs to the other tetracyclines, including eravacycline and omadacycline, while the putative ancestral gene *fmo* was inactive.

**Fig. 3:**
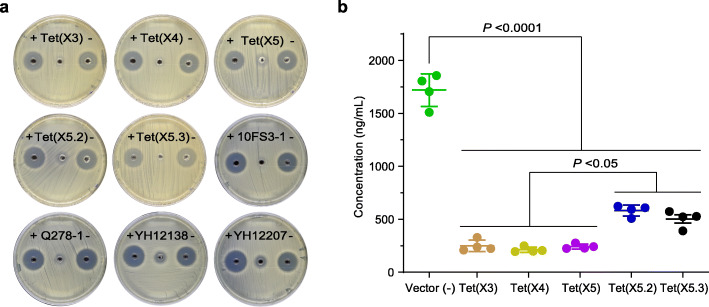
Comparative analysis of *tet*(X)-mediated eravacycline degradation. **a** Microbiological degradation for eravacycline. Five pBAD24-*tet*(X) constructs as well as their parental strains, including Clade_U6 10FS3-1 [*tet*(X3)-positive], *A. indicus* Q278-1 [*tet*(X4)-positive], Clade_U1 YH12138 [*tet*(X3)- and *tet*(X5.2)-positive], and *A. piscicola* YH12207 [*tet*(X3)- and *tet*(X5.3)-positive], are used. The presence of Tet(X) degrades eravacycline, and consequently, the degraded eravacycline loses its antimicrobial activity against a susceptible indicator strain. By contrast, the absence of Tet(X) yields a clear inhibition zone, with a diameter of > 18 mm. The groups with the addition of untreated eravacycline or with the addition of eravacycline treated with *E. coli* JM109 carrying the empty vector pBAD24 serve as negative controls. **b** LC-MS/MS quantification of eravacycline degradation by *tet*(X) clones. Individual values of four biological replicates are shown as dots, while the means and standard deviations are displayed as error bars. Vector (−), the control group with the addition of eravacycline treated with pBAD24-carrying *E. coli* JM109

Microbiological degradation assays revealed that these five *tet*(X) clones as well as their parental strains could effectively inactivate eravacycline (Fig. 3a). Notably, qRT-PCR revealed that the transcript expression levels of chromosomal *tet*(X5.2) in *Acinetobacter* clade_U1 YH12138 and plasmid-mediated *tet*(X5.3) in *A. piscicola* YH12207 were (17.6 ± 3.2)% and (83.3 ± 4.9)%, respectively, when compared with that of the coexisting *tet*(X3). The eravacycline inactivation effects were further confirmed by LC-MS/MS. The results showed the eravacycline concentrations had (70.8 ± 2.2)% reduction in Tet(X5.2) and (74.8 ± 3.4)% reduction in Tet(X5.3) constructs, respectively (Fig. 3b). Similar eravacycline degradation efficiencies were observed in the control Tet(X3) [(87.5 ± 2.3)%], Tet(X4) [(89.4 ± 1.1)%], and Tet(X5) [(87.9 ± 1.0)%] constructs (Fig. 3b). These results indicated that *tet*(X5.2) and *tet*(X5.3) genes were also able to degrade tetracycline antibiotics, thereby conferring to high-level resistance.

In total, 20.7% (40/193) of the *tet*(X)-positive *Acinetobacter* spp. isolates were found to carry two different *tet*(X) variants. Specifically, 18.7% (36/193) of the *tet*(X3)-positive (*n* = 34) and *tet*(X4)-positive (*n* = 2) *Acinetobacter* spp. strains were found to co-harbor *tet*(X5.2), which were collected from various sample sources in nine provinces (Fig. 1; Additional file 1: Table S2). In addition, 2.1% (4/193) of isolates were found to co-harbor *tet*(X5.3) and *tet*(X3). They were all from *A. piscicola* and isolated from pig (*n* = 2) and soil (*n* = 2) samples in Zhejiang province. None of the isolates was found to co-harbor other or more than two *tet*(X) variants.

### Antimicrobial resistance profile of *tet*(X)-carrying *Acinetobacter* spp. isolates

Susceptibility testing showed that all *tet*(X)-carrying *Acinetobacter* spp. strains were resistant to tigecycline and tetracycline, and exhibited high MIC levels to eravacycline (2–8 μg/mL) and omadacycline (8–16 μg/mL). These isolates were commonly resistant to trimethoprim/sulfamethoxazole (91.2%; 176/193), florfenicol (88.1%; 170/193), and ciprofloxacin (69.9%; 135/193), but less frequently resistant to other antibiotics (namely gentamicin, ampicillin, amikacin, colistin, meropenem, and cefotaxime), with the resistance rates ranging from 0.5 to 24.9% (Additional file 1: Figure S3). Among the major species, Clade_U1 showed higher ciprofloxacin (83.1%) and ampicillin (40.0%) resistance rates, while Clade_U2 exhibited 100% florfenicol resistance. Additionally, 53.3% of *A. lwoffii* isolates showed gentamicin resistance. In silico resistance gene mining from their whole-genome sequences showed that the *tet*(X) genes were commonly associated with *sul2* and *floR* (Fig. 2c), which correlated with the trimethoprim/sulfamethoxazole and florfenicol resistance phenotypes described above. Notably, one *A. indicus* isolate (0.5%; 1/193) was resistant to meropenem and cefotaxime. A further WGS analysis identified the presence of carbapenem-resistant gene *bla*_NDM-1_ and *tet*(X3) on the same 3.2-Mb chromosome.

### *tet*(X)*-*harboring plasmid and chromosome sequences

**Fig. 4:**
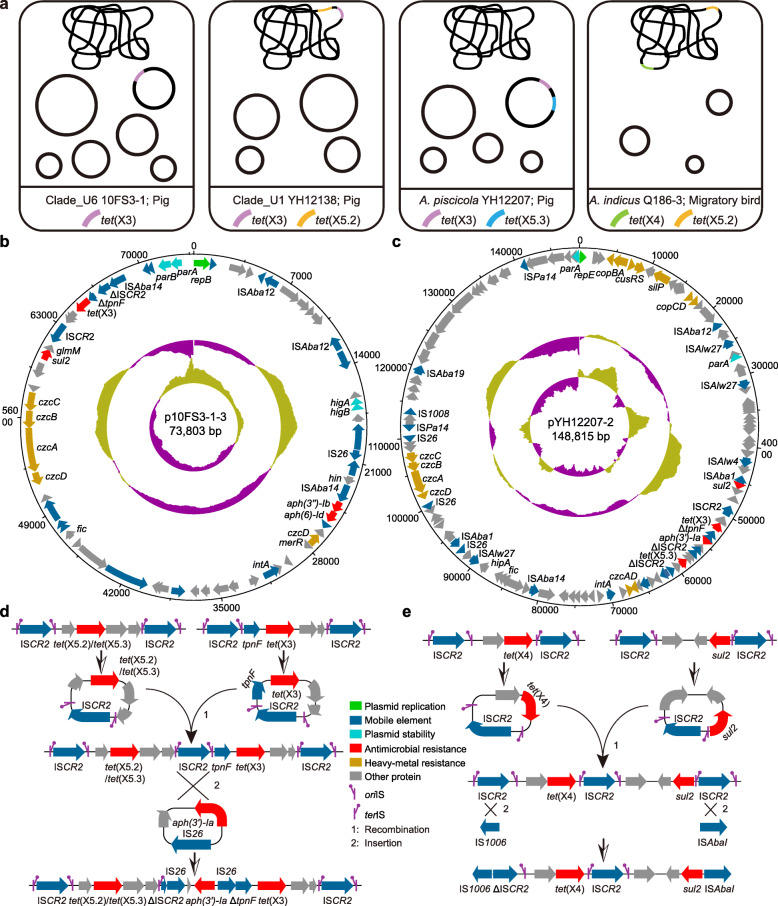
Genetic structures of *tet*(X)-positive plasmids and chromosomes. **a** Sketch maps of the *tet*(X3)-, *tet*(X4)-, *tet*(X5.2)-, and *tet*(X5.3)-positive *Acinetobacter* spp. strains. Bacterial species, isolation sources, and *tet*(X) variants are illustrated. **b**, **c** Structures of the *tet*(X3)-harboring plasmid p10FS3-1-3 (**b**) and the *tet*(X3)- and *tet*(X5.3)-co-harboring plasmid pYH12207-2 (**c**). GC skew and GC content are indicated from the inside out. The arrows represent the positions and transcriptional directions of the ORFs. Δ symbol indicates the truncated gene. **d**, **e** Putative integration processes of *tet*(X)-carrying composite transposons in Clade_U1 YH12138 (**d**), *A. piscicola* YH12207 (**d**), and *A. indicus* Q186-3 (**e**). The replication initiation site *ori*IS and replication termination site *ter*IS of the IS*CR2* element are also indicated

The combination of Nanopore and HiSeq genomic assembly obtained the complete *tet*(X)-carrying plasmid and chromosome sequences from several representative *Acinetobacter* spp. strains (Fig. 4a). However, these *tet*(X)-positive plasmid sequences showed low nucleotide sequence identities (< 70%) between each other, suggesting the spread of *tet*(X) is not due to the horizontal transfer of a predominant plasmid.

For the *Acinetobacter* Clade_U6 strain 10FS3-1, the *tet*(X3) gene was located on a 73,803-bp untypable plasmid, p10FS3-1-3. p10FS3-1-3 had an average GC content of 42.5% and harbored 61 putative ORFs, including genes encoding resistance to sulfonamide (*sul2*), aminoglycosides [*aph(3″)-Ib, aph(6)-Id*], and heavy metals (*czcA*-*czcD*) (Fig. 4b). For the *A. piscicola* strain YH12207, the *tet*(X3) and *tet*(X5.3) genes formed a composite transposon-like structure of ΔIS*CR2*-hp-*tet*(X5.3)-hp-hp-ΔIS*CR2*-IS*26*-hp-*aph(3′)-Ia*-IS*26*-Δ*tpnF*-*tet*(X3)-hp-hp-IS*CR2* and were localized on an 148,815-bp untypable plasmid pYH12207-2, possessing 128 ORFs with a GC content of 39% (Fig. 4c). Similarly, the *tet*(X3) and *tet*(X5.2) genes were located on the chromosome of the *Acinetobacter* clade_U1 strain YH12138, carrying a similar composite transposon-like structure of ΔIS*CR2*-hp-*tet*(X5.2)-hp-hp-ΔIS*CR2*-IS*26*-hp-*aph(3′)-Ia*-IS*26*-Δ*tpnF*-*tet*(X3)-hp-hp-IS*CR2*. Furthermore, *tet*(X4) and *tet*(X5.2) were found on the chromosome of *A. indicus* Q186-3 at different regions.

Conjugation experiments showed that both p10FS3-1-3 [*tet*(X3)-positive] and pYH12207-2 [*tet*(X3)- and *tet*(X5.3)-positive] could be successfully transferred into the recipient strain *A. baylyi* ADP1 at a low frequency of ~ 10^−9^, confirming the conjugability of *tet*(X)-harboring plasmids. There was no significant additive effect on MICs of tetracycline antibiotics between pYH12207-2 and p10FS3-1-3 transconjugants (Additional file 1: Table S3). Moreover, the conjugation experiments failed despite multiple attempts when Enterobacteriaceae bacteria were used as recipient strains, suggesting these plasmids could only replicate in *Acinetobacter* species.

### Genetic environments of *tet*(X) variants

Previous studies showed that the *tet*(X) variants were usually associated with an IS*91*-like element IS*CR2*, and some typical transposon structures IS*CR2*-*tpnF*-*tet*(X3)-hp-hp-IS*CR2* for *tet*(X3), IS*CR2*-*catD*-*tet*(X4)-IS*CR2* for *tet*(X4), and IS*CR2*-*tpnF*-*tet*(X5)-hp-hp-IS*CR2* for *tet*(X5) were described [5–7]. Similarly, the genetic analyses of *tet*(X3), *tet*(X4), *tet*(X5.2), and *tet*(X5.3) in this study revealed the same close association between IS*CR2* and different *tet*(X) variants. Intact or partial IS*CR2* sequences were identified in the four *tet*(X) variants among most isolates (Additional file 1: Figure S4).

Two major *tet*(X3) genetic structures were identified in 187 *tet*(X3)-positive *Acinetobacter* spp. strains [one with a truncated *tet*(X3) was not included; Additional file 1: Figure S4a]. The previously described IS*CR2*-*tpnF*-*tet*(X3)-hp-hp-IS*CR2* gene cassette was found in 38.0% (71/187) of *Acinetobacter* isolates, including two isolates with partial sequence deletion between *tpnF* and *tet*(X3) (types II and III). In the rest 115 (61.5%) *tet*(X3)-harboring isolates, the recombinase gene *tpnF* that located upstream of *tet*(X3) was truncated by an IS*26* insertion (type I).

Similarly, the same IS*CR2* element was found downstream of *tet*(X4), *tet*(X5.2), and *tet*(X5.3), respectively, but additional genetic rearrangements and IS insertion were identified (Additional file 1: Figure S4b-4c). It was noteworthy that the *tet*(X4)-harboring contigs in four *Acinetobacter* spp. strains showed 100% sequence identities to the gene cassette of IS*CR2*-*catD*-*tet*(X4)-IS*CR2* that we previously described in Enterobacteriaceae and *Aeromonas caviae* strains (Additional file 1: Figure S4b). For *tet*(X5) variants, *tet*(X5.2) and *tet*(X5.3), 80.0% (32/40) of them shared a similar cassette as the structure in *E. coli* 912, *Proteus cibarius* ZF2, and *Chryseobacterium* spp. BGARF1 strains (Additional file 1: Figure S4c). The close association between IS*CR2* and different *tet*(X) variants, and the high sequence identities of *tet*(X) cassette from difference species further highlighted the critical role of IS*CR2* in the dissemination of *tet*(X).

The IS*CR2*-mediated *tet*(X) transposition was further experimentally confirmed using the *tet*(X3) cassette [Δ*tpnF*-*tet*(X3)-hp-hp-IS*CR2*] cloned from Clade_U6 10FS3-1. The *tet*(X3) cassette was successfully transferred to the recipient strains *A. baylyi* ADP1 and *E. coli* MG1655 at a frequency of (9.6 ± 2.7) × 10^−6^ and (5.4 ± 1.4) × 10^−8^, respectively, which was consistent with our previous report for *tet*(X4) in *A. caviae* [11].

### Molecular evolutionary analysis of Tet(X)

**Fig. 5:**
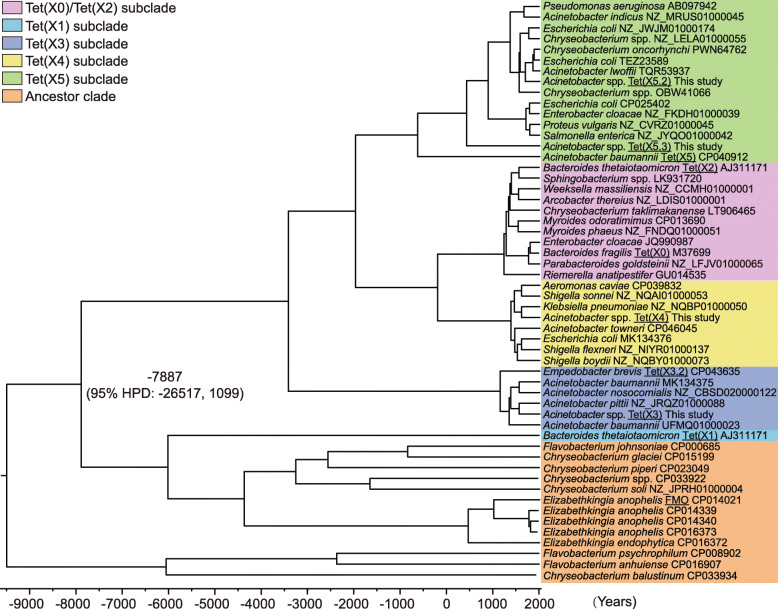
Bayesian phylogenetic inference of Tet(X) proteins by relaxed clock model. The reported Tet(X) variants are underlined, while different Tet(X) subclades are shown by shading in different colors. The divergence time as well as its 95% HPD between Tet(X) and monooxygenase subclades is shown around the node. The tip labels are annotated by bacterial species and their corresponding GenBank accession numbers

The phylogenetic reconstruction showed that Tet(X) variants, Tet(X0)–Tet(X5), and their closely related sequences formed a separate subclade (Fig. 5). Among them, Tet(X0) [first described as Tet(X) in *Bacteroides fragilis* [36], and tentatively designated as Tet(X0) in this study to differentiate from other Tet(X) variants], Tet(X1), and Tet(X2) were the earliest identified Tet(X) enzymes, but Tet(X1) was nonfunctional due to its N-terminal truncation [8]. The sequences in Tet(X3), Tet(X4), and Tet(X5) subclades showed approximate 85%, 95%, and 87% amino acid sequence identities to those in the Tet(X0)/Tet(X2) subclade, respectively. Notably, the Tet(X0)/Tet(X2) subclade mainly consisted of anaerobic *Bacteroides* spp. and *Riemerella anatipestifer*, while the Tet(X3), Tet(X4), and Tet(X5) subclades consisted of aerobic *Acinetobacter* spp. and Enterobacteriaceae bacteria.

By contrast, the chromosomal monooxygenases from several environmental Flavobacteriaceae bacteria, including *Chryseobacterium* spp. (e.g., CP015199), *Flavobacterium* spp. (e.g., CP000685), and *Elizabethkingia* spp. (e.g., CP016372), shared an approximate 55.8% sequence identity to the first reported Tet(X0). A Bayesian evolutionary analysis indicated the divergence time of Tet(X) clades [except the truncated Tet(X1)] from the chromosomal monooxygenases likely occurred ~ 9900 years ago [7887 BC; 95% highest probability density (HPD), 26,517 BC–AD 1099].

The average GC content of Tet(X) variants (approximate 37%) was similar to the average genomic GC content of Flavobacteriaceae bacteria (33–38%), but much lower than that of *Acinetobacter* spp. and Enterobacteriaceae bacteria (> 45%). These results suggested that the environmental Flavobacteriaceae-related bacteria constitute a putative ancestral subclade, leading to the recent emergence of *tet*(X)-like genes in *Acinetobacter* spp. and Enterobacteriaceae (Additional file 1: Figure S5).

## Discussion

One of the most significant findings in this study was the high prevalence of *tet*(X) genes among *Acinetobacter* spp. strains from different sources, especially in pig farms (7.3%, 187/2575), which was considerably higher than what we previously reported for *E. coli* from samples in pigs and their surrounding environments (1.1%, 34/2970) [6]. The results suggested that *tet*(X)-harboring *Acinetobacter* spp. strains have been widely spread in animal and environment sources from different regions in China. Importantly, these *tet*(X)-harboring *Acinetobacter* spp. strains were broadly dispensed into 12 species, including six putative novel species that were firstly described here. The six novel species covered over 50% of the *tet*(X)-positive *Acinetobacter* spp. isolates identified in this study. The results further indicated that our understanding about the bacterial hosts for *tet*(X) was limited, and previous studies might significantly underestimate the paramount role of *Acinetobacter* species in the dissemination of *tet*(X)-mediated tigecycline resistance.

The *Acinetobacter* spp. strains are ubiquitous in the natural environment [37], and we suspect that these *tet*(X)-positive *Acinetobacter* strains, including isolates from the novel species (Clade_U1 to U6), likely originate from environmental sources. Although tigecycline is only approved for clinical settings, the other tetracycline antibiotics, including tetracycline, oxytetracycline, and chlortetracycline, were heavily utilized in animal and agricultural production [38, 39]. The selection pressure exacted by them might promote the horizontal transfer of *tet*(X) into *Acinetobacter* spp. strains. Similarly, some other antimicrobials (e.g., sulfonamides, phenicols, and quinolones) were also frequently used in animal feed in China, which were consistent with our > 65% resistance rates for trimethoprim/sulfamethoxazole, florfenicol, and ciprofloxacin in these *tet*(X)-positive *Acinetobacter* spp. isolates (Additional file 1: Figure S3).

Although *tet*(X)-positive *Acinetobacter* spp. strains are universal in animal and environmental sources, some of the *Acinetobacter* species (e.g., *A. lwoffii* and *A. schindleri*) are opportunistic pathogens and have been identified as sources of nosocomial infections, including septicemia, pneumonia, meningitis, urinary tract infections, and skin and wound infections [40, 41]. In this study, we detected a colonized *tet*(X3)-positive *A. lwoffii* isolate (YH18001) from the urine sample in a healthy person undergoing physical examination. Similarly, previous studies also reported the identification of *tet*(X)-harboring *A. baumannii* isolates from clinical specimens among inpatients [5, 7]. These results suggested *tet*(X) genes have disseminated into pathogenic *Acinetobacter* species, which might render tigecycline ineffective and severely limited therapeutic options. Worrisomely, some *tet*(X)-positive *Acinetobacter* strains also harbored additional resistance mechanisms, such as carbapenemase gene *bla*_NDM-1_ (e.g., *A. indicus* C15), conferring resistance to clinically important antimicrobials (e.g., cephalosporins and carbapenems) [42, 43].

The high diversity of *Acinetobacter* species harboring *tet*(X) variants across China further suggested that the wide distribution of *tet*(X) is mediated by horizontal genetic transfer rather than a single predominant bacterial species or clone, which was also supported by the finding of low sequence identities between different *tet*(X)-harboring plasmids (e.g., p10FS3-1-3 and pYH12207-2). In addition to *tet*(X3) and *tet*(X4), two *tet*(X) variants *tet*(X5.2) and *tet*(X5.3) were detected in this study, with conservative genetic environments across 12 *Acinetobacter* species in human, pig, migratory bird, soil, dust, sewage, and vegetable samples. Our gene construct and phenotypic work also confirmed the high-level enzymatic degradation ability and tigecycline resistance that conferred by them.

Interestingly, 40 (20.7%) isolates harbored two different *tet*(X) variants. The molecular mechanism underlying the genetic redundancy of *tet*(X) remained poorly understood, which might be in part explained by the repeated acquisitions of *tet*(X) genes through IS*CR2*-mediated transposition. Thus far, all *tet*(X) variants conferring high-level tigecycline resistance have been linked to IS*CR2*, which shows the ability to mobilize various resistance genes (e.g., *rmtH* and *bla*_VEB_) by rolling-circle transposition [44, 45]. Our current and previous studies also confirmed that IS*CR2* could transfer *tet*(X) in *Acinetobacter* spp. and *A. caviae* strains via transposition [11]. Accordingly, the composite structures of *tet*(X) genes in *Acinetobacter* strains might be generated randomly by the IS*CR2*-mediated transposition and recombination (Fig. 4d, e), as previously reported for the tandem structure of *tet*(X4) [5]. It should be noted that these genetic structures were found on both plasmid and chromosome in *Acinetobacter* species, highlighting that IS*CR2* was highly active in mobilizing *tet*(X)-mediated tigecycline resistance. However, the presence of more than one *tet*(X) variants did not seem to confer additive resistance to tetracyclines (Additional file 1: Table S3). We suspected that the relative stability of *tet*(X)-mediated tigecycline resistance may result from the balance between *tet*(X) expression and bacterial survival, which warranted further studies [46].

Previous studies indicated that IS*CR2* was highly dispersed among *Acinetobacter* spp. strains [47]. Genomic examination of 5433 *Acinetobacter* spp. draft genome sequences in the NCBI database revealed that 54.5% (*n* = 2960) of *Acinetobacter* genomes contained IS*CR2* [48]. Similarly, 17.7% (*n* = 3333) of 18,815 draft *E. coli* genomes were found to harbor IS*CR2*. However, the frequency of IS*CR2* in Flavobacteriaceae bacteria, including *Chryseobacterium* spp., *Flavobacterium* spp., and *Elizabethkingia* spp., was much lower (0.6%, 5/830). The results suggested that, in comparison to Flavobacteriaceae species, IS*CR2* (and its neighboring sequences) has a higher propensity to be integrated in the *Acinetobacter* and *E. coli* genomes, which might partially explain the higher detection rates of *tet*(X) among these species.

Our phylogenetic reconstruction indicated that the *tet*(X) variants shared the same most recent common ancestor as the chromosome-borne monooxygenase genes in Flavobacteriaceae bacteria, and the divergence between them occurred at ~ 7887 BC, although a potential polyphyletic relationship could not be completely ruled out. We hypothesized that the molecular evolution of *tet*(X) started with the mobilization of *tet*(X)-like monooxygenase sequences (or along with their neighboring genes) by IS*CR2*-like transposase gene from the chromosome of Flavobacteriaceae species over ~ 9900 years ago, followed by the integration into some environmental bacterial genomes (e.g., *Acinetobacter* spp. or *E. coli*). IS*CR2* further mobilized the *tet*(X) cassette into different plasmid or chromosome locations through transposition due to its active nature among these new hosts. The divergence of *tet*(X) variants pre-dated the discovery and clinical use of tetracycline antibiotics. Under the antibiotic selection pressure, the *tet*(X) variants were selected and subsequently transferred through conjugation or other transfer mechanisms. Our conjugation and transposition experiments also confirmed the transferability of *tet*(X)-harboring plasmids and transposon. The similar mechanism has been proposed to interpret the molecular evolution of *mcr-1* [49].

## Conclusions

Taken together, our study reported a significantly high prevalence of *tet*(X) genes in *Acinetobacter* spp. strains across China from various sources. In addition to *tet*(X3) and *tet*(X4), *tet*(X5.2) and *tet*(X5.3) were also confirmed to have high-level degradation activities on tetracyclines but without additive effects between different variants. These *tet*(X) genes might be further spread by plasmids and the IS*CR2* element. Worrisomely, the *tet*(X)-mediated tigecycline resistance has been detected in carbapenem- and colistin-resistant *Acinetobacter* spp. strains. Future efforts are needed to improve the surveillance of *tet*(X) genes from all related sectors, and to monitor the occurrence of *tet*(X) in clinical pathogens and evaluate the clinical impacts.

## Supplementary Information

**Additional file 1: Figure S1.** Sequence alignment of Tet(X) variants by ESPript version 3.0. **Figure S2.** Structural characteristics of Tet(X5)-like proteins. **Figure S3.** Resistance rates of the *tet*(X)-positive *Acinetobacter* spp. strains against 11 antibiotics. **Figure S4.** Comparative analysis of the *tet*(X)-carrying structures. **Figure S5.** Hypothesized evolution model of *tet*(X) genes. **Table S1.** Primers used in this study. **Table S2.** Prevalence of *tet*(X) genes in *Acinetobacter* spp. strains by origin. **Table S3.** MICs of tetracyclines for the studied strains.

## Data Availability

The whole-genome sequencing data of 193 *tet*(X)-positive *Acinetobacter* spp. strains sequenced by Illumina HiSeq are available in the NCBI BioProject repository under the accession number PRJNA558522 [50]. The complete genomic sequences of *Acinetobacter* Clade_U1 YH12138, *A. piscicola* YH12207, *A. indicus* Q186-3, *A. indicus* C15, and *Acinetobacter* Clade_U6 10FS3-1 have also been deposited under PRJNA604696 [51] and PRJNA531082 [52], respectively. The accession numbers of all the other sequences analyzed during the current study are included in this published article and available in the NCBI Nucleotide or Protein database [53].

## Abbreviations

ANI: Average nucleotide identity
CLSI: Clinical and Laboratory Standards Institute
CRAB: Carbapenem-resistant *Acinetobacter baumannii*
CRE: Carbapenem-resistant Enterobacteriaceae
ERIC-PCR: Enterobacterial repetitive intergenic consensus PCR
ESS: Effective sample size
HPD: Highest probability density
IACUC: Institutional Animal Care and Use Committee
LB: Luria-Bertani
LC-MS/MS: Liquid chromatography-tandem mass spectrometry
MDR: Multidrug-resistant
MH: Mueller-Hinton
MICs: Minimum inhibitory concentrations
MRSA: Methicillin-resistant *Staphylococcus aureus*
NCBI: National Center for Biotechnology Information
ORFs: Open reading frames
qRT-PCR: Quantitative reverse transcription-PCR
USFDA: United States Food and Drug Administration
VRE: Vancomycin-resistant *Enterococcus*
WGS: Whole-genome sequencing
XDR: Extensively drug-resistant
