# Supplementary material for: Genetic diversity and characteristics of high-level tigecycline resistance Tet(X) in Acinetobacter species
Source: Genome Med. 2020 Dec 7;12:111. doi: 10.1186/s13073-020-00807-5 (PMC7722449; doi:10.1186/s13073-020-00807-5)
Supplement: Supplementary file 1 — Additional file 1: Figure S1. Sequence alignment of Tet(X) variants by ESPript version 3.0. Figure S2. Structural characteristics of Tet(X5)-like proteins. Figure S3. Resistance rates of the tet(X)-positive Acinetobacter spp. strains against 11 antibiotics. Figure S4. Comparative analysis of the tet(X)-carrying structures. Figure S5. Hypothesized evolution model of tet(X) genes. Table S1. Primers used in this study. Table S2. Prevalence of tet(X) genes in Acinetobacter spp. strains by origin. Table S3. MICs of tetracyclines for the studied strains. [file 13073_2020_807_MOESM1_ESM.doc]

**Supplementary information for**

**Title: Genetic diversity and characteristics of high-level tigecycline resistance Tet(X) in *Acinetobacter* species**

Chong Chen^1,2,3^, Chao-Yue Cui^1,2,3^, Jun-Jun Yu^4^, Qian He^1,2,3^, Xiao-Ting Wu^1,2,3^, Yu-Zhang He^1,2,3^, Ze-Hua Cui^1,2,3^, Cang Li^1,2,3^, Qiu-Lin Jia^1,2,3^, Xiang-Guang Shen^3^, Ruan-Yang Sun^1,2,3^, Xi-Ran Wang^1,2,3^, Min-Ge Wang^1,2,3^, Tian Tang^1,2,3^, Yan Zhang^1,2,3^, Xiao-Ping Liao^1,2,3^, Barry N. Kreiswirth^5^, Shi-Dan Zhou^6^, Bin Huang^7^, Hong Du^8^, Jian Sun^1,2,3*^, Liang Chen^5,9*^ and Ya-Hong Liu^1,2,3*^

^1^National Risk Assessment Laboratory for Antimicrobial Resistance of Animal Original Bacteria, College of Veterinary Medicine, South China Agricultural University, Guangzhou, China.

^2^Guangdong Laboratory for Lingnan Modern Agriculture, South China Agricultural University, Guangzhou, China.

^3^Guangdong Provincial Key Laboratory of Veterinary Pharmaceutics Development and Safety Evaluation, South China Agricultural University, Guangzhou, China.

^4^Guangdong Enterprise Key Laboratory for Animal Health and Environmental Control, WENS Foodstuff Group Co Ltd, Xinxing, China.

^5^Center for Discovery and Innovation, Hackensack Meridian Health, Nutley, NJ, USA.

^6^Intensive Care Unit, Huizhou Municipal Central Hospital, Huizhou, China.

^7^Department of Laboratory Medicine, The First Affiliated Hospital of Sun Yat-sen University, Guangzhou, China.

^8^Department of Clinical Laboratory, The Second Affiliated Hospital of Soochow University, Suzhou, China.

^9^Hackensack Meridian School of Medicine at Seton Hall University, Nutley, NJ, USA.

**
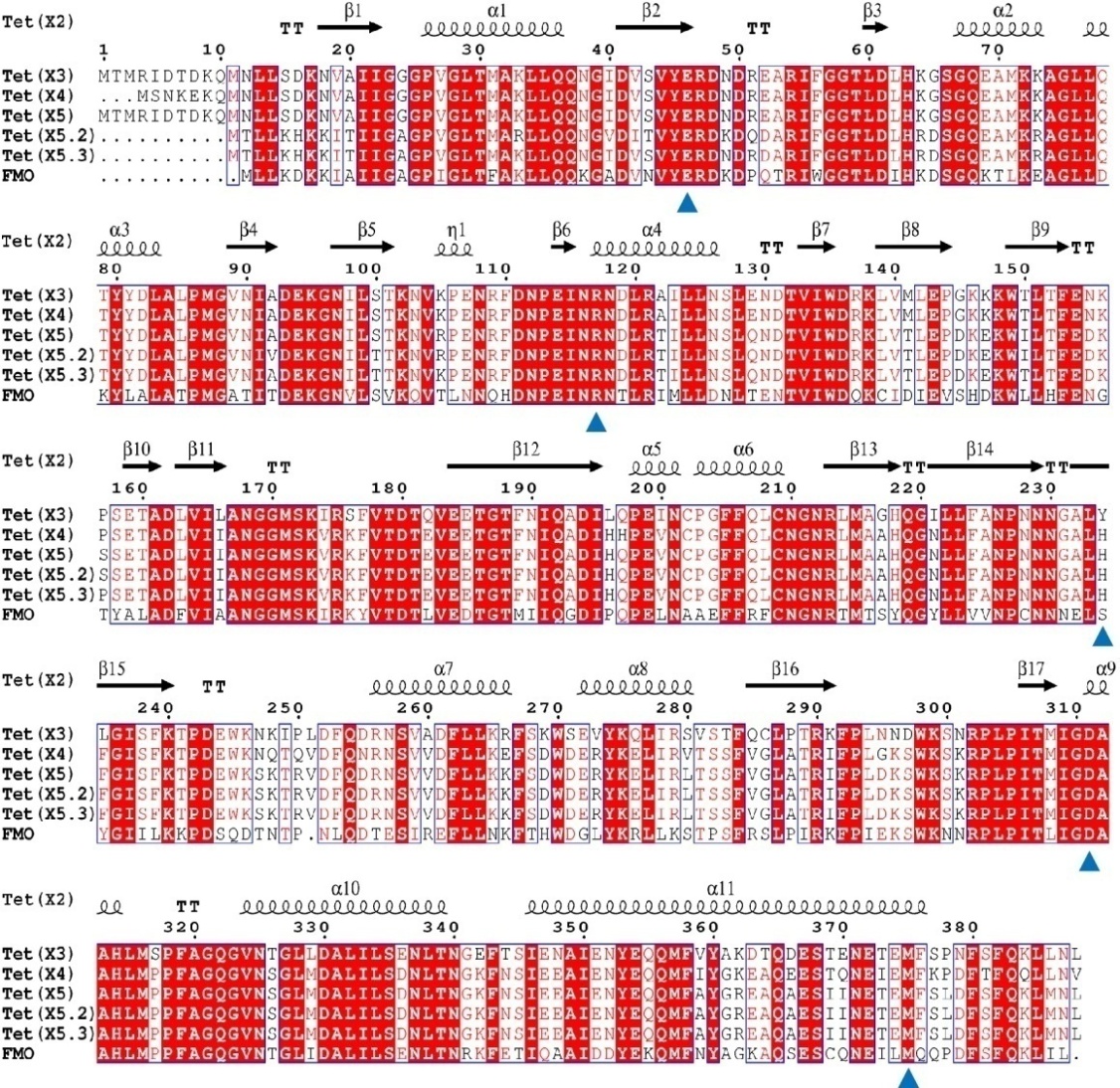
**

**Fig. S1** Sequence alignment of Tet(X) variants by ESPript version 3.0 . Secondary structure elements of Tet(X2) (PDB accession number: 2XDO) are presented on top, with squiggles for alpha helices, arrows for beta sheets and TT letters for random coils. The reported key amino acid sites of Tet(X) are also indicated by triangles in blue .

**
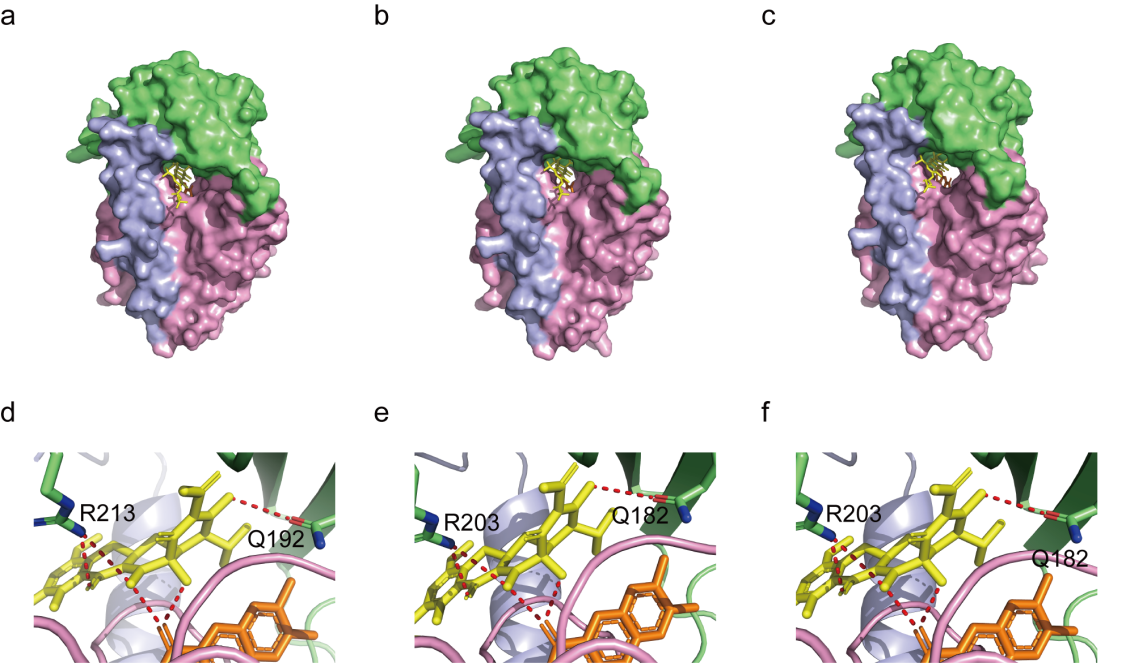
**

**Fig. S2** Structural characteristics of Tet(X5)-like proteins. Homology modelling of Tet(X5) (**a**), Tet(X5.2) (**b**) and Tet(X5.3) (**c**) are generated from SWISS-MODEL, with Tet(X2) (PDB: 4A6N) as the template . Meanwhile, the polar contacts of Tet(X5) (**d**), Tet(X5.2) (**e**) and Tet(X5.3) (**f**) with tigecycline are also analyzed and marked by dotted lines in red. The substrate-binding domain (light green), FAD-binding domain (pink), C-terminal α-helix (light blue), substrate tigecycline (yellow) and ligand FAD (orange) are displayed, respectively.


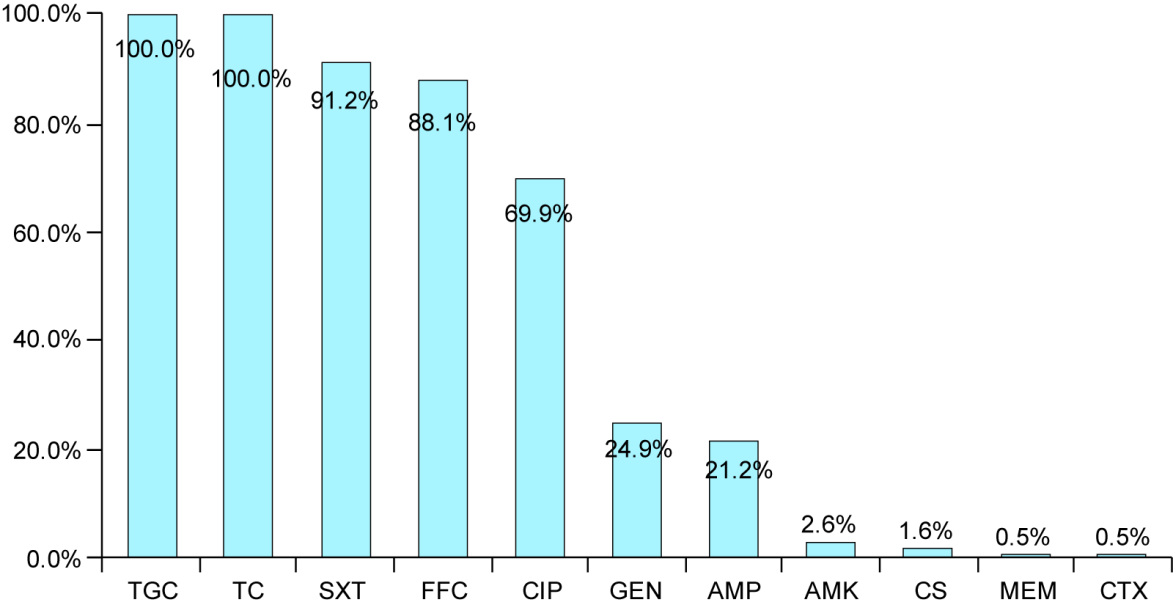


**Fig. S3** Resistance rates of the *tet*(X)-positive *Acinetobacter* spp. strains against 11 antibiotics. The resistance rates are indicated on the top of different antibiotics. TGC, tigecycline; TC, tetracycline; SXT, trimethoprim/sulfamethoxazole; FFC, florfenicol; CIP, ciprofloxacin; GEN, gentamicin; AMP, ampicillin; AMK, amikacin; CS, colistin; MEM, meropenem; CTX, cefotaxime.


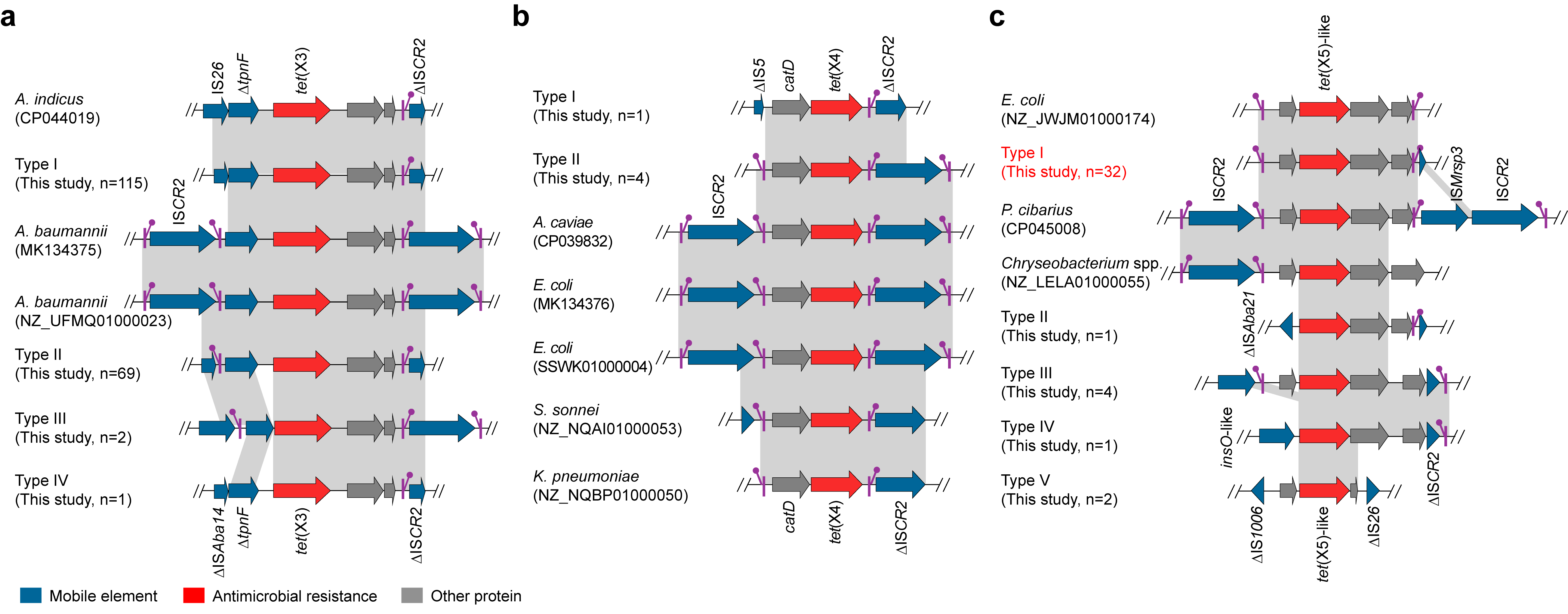
**Fig. S4** Comparative analysis of the *tet*(X)-carrying structures. Genetic environments of *tet*(X3) (**a**), *tet*(X4) (**b**), *tet*(X5.2) (**c**) and *tet*(X5.3) (**c**) genes are analyzed, respectively. For *tet*(X5.3)-positive strains, all of them (n=4) share a similar environment with type I for *tet*(X5.2) (n=28) in this study, and therefore are combined and indicated in red. Regions of >99% nucleotide sequence homology are marked by grey shading.

**
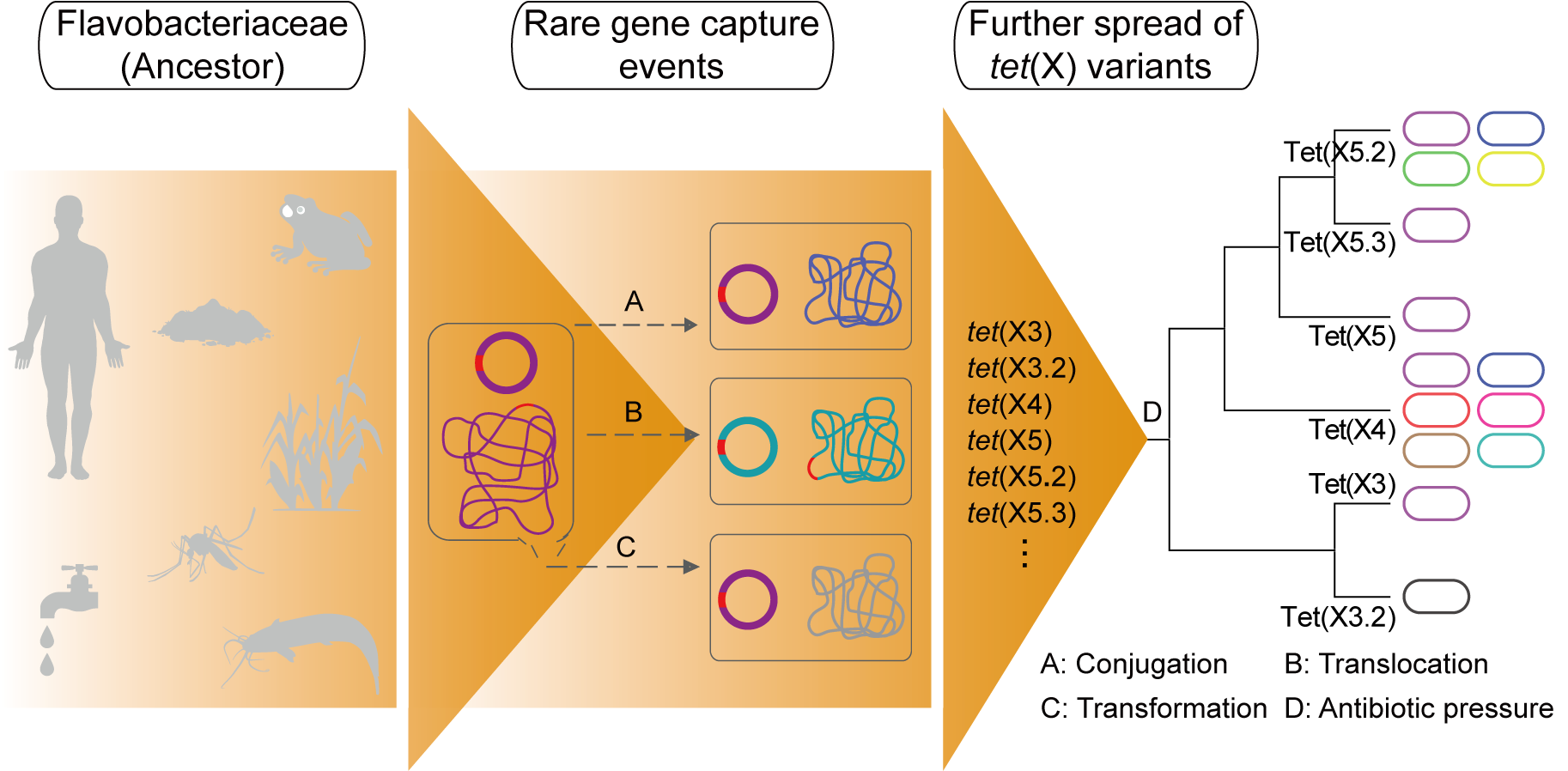
**

**Fig. S5** Hypothesized evolution model of *tet*(X) genes. The ancestral *tet*(X)-like gene is located on the chromosome of environmental Flavobacteriaceae bacteria. Rare gene capture events mediated by mobile genetic elements (e.g., IS*CR2*-like) mobilize the *tet*(X)-like gene from the chromosome to conjugative plasmids or transposons, followed by horizontal transfer to other species by conjugation (**A**) or translocation (**B**). Uptake of free DNA by competent bacterial cell (**C**) may also occur. Under the antibiotic selection pressure (**D**), these *tet*(X) genes further spread into other bacterial hosts, such as *Acinetobacter* spp. (depicted as an ellipse in purple), *E. coli* (blue), *P. cibarius* (green), *Chryseobacterium* spp. (yellow), *K. pneumoniae* (red), *Shigella* spp. (fuchsia), *Salmonella* spp. (brown), *A. caviae* (cyan) and *E. brevis* (black).

**Table S1 Primers used in this study.**

| Primers | Nucleotide sequences (5’ to 3’)^a^ | Sizes |
| --- | --- | --- |
| *tet*(X3)-F | GAGCTTTGTTACCGACACGC | 584 bp |
| *tet*(X3)-R | CTGTCGATTCGTCCTGCGTA |
| *tet*(X4)-F | CCCGTCACTCCCACTCAATAG | 1523 bp |
| *tet*(X4)-R | TTGGCACTATAAAAACGCTGGT |
| *tet*(X3)-EcoRI | TACGCGAATTCATGACAATGCGAATAGATAC | 1167 bp |
| *tet*(X3)-SalI | TACGCGTCGACTTATAGATTCAATAATTTTTGAAAC |
| *tet*(X4)-EcoRI | TACGCGAATTCATGAGCAATAAAGAAAAACAAATGAATTTAC | 1158 bp |
| *tet*(X4)-SalI | TACGCGTCGACTTATACATTTAACAATTGCTGAAACG |
| *tet*(X5)-EcoRI | TACGCGAATTCATGACAATGCGAATAGATACAGAC | 1167 bp |
| *tet*(X5)-SalI | TACGCGTCGACTTATAGATTCATTAGTTTTTGGAAAGAAAAGTCG |
| *tet*(X5.2)-EcoRI | TACGCGAATTCATGACTTTACTAAAACATAAAAAAA | 1137 bp |
| *tet*(X5.2)-SalI | TACGCGTCGACTTATAGATTCATTAGTTTTTGGAAA |
| *tet*(X5.3)-EcoRI | TACGCGAATTCATGACTTTATTAAAACATAAAAAAATTACAATAATTGG | 1137 bp |
| *tet*(X5.3)-SalI | TACGCGTCGACTTATAGATTCATTAGTTTTTGGAACG |
| *fmo*-SbfI | TACGCCCTGCAGGATGTTACTAAAAGATAAAAAAATAGCCATTATCGG | 1128 bp |
| *fmo*-HindIII | TACGCAAGCTTCTATAGGATTAACTTCTGAAATGAGAAATCAGGTTGTTGC |
| *tet*(X5.2)-PstI^b^ | TACGCCTGCAGATGACTTTACTAAAACATAAAAAAA | 1137 bp |
| *tet*(X5.2)-HindIII^b^ | TACGCAAGCTTTTATAGATTCATTAGTTTTTGGAAA |
| *tet*(X5.3)-PstI^b^ | TACGCCTGCAGATGACTTTATTAAAACATAAAAAAATTACAATAATTGG | 1137 bp |
| *tet*(X5.3)-HindIII^b^ | TACGCAAGCTTTTATAGATTCATTAGTTTTTGGAACG |
| *tet*(X3)-qF | CAGGACAGAAACAGCGTTGC | 179 bp |
| *tet*(X3)-qR | GCAGCATCGCCAATCATTGT |
| *tet*(X5.2)/*tet*(X5.3)-qF | AAGCGTCCATTACCCATAACGA | 200 bp |
| *tet*(X5.2)/*tet*(X5.3)-qR | TCTGCCTGTGCTTCTCTTCCA |
| 16S rRNA-qF | ATTAGATACCCTGGTAGTCCACGC | 316 bp |
| 16S rRNA-qR | TTGCGGGACTTAACCCAAC |
| pBAD24-HF | CGGCGTCACACTTTGCTATG | 1455 bp |
| pBAD24-HR | ACGGCGTTTCACTTCTGAGT |
| *tet*(X3)-HF | AGACGGAGACTTAAGGCGTACGTCGATCGATTGAATGAAGGAC | 4904 bp |
| *tet*(X3)-HR | GGAGATCTACTACTGTGGCTATATATGCTTGAACCCGCGCT |

^a^Restriction sites or homologous sequences that used for molecular cloning are underlined.

^b^These primers are used for cloning based on the recombinant plasmid pBAD24+*tet*(X3).

**Table S2** Prevalence of *tet*(X) genes in *Acinetobacter* spp. strains by origin.

| Sources | Samples | *tet*(X3) | *tet*(X4) | *tet*(X5.2) | *tet*(X5.3) |
| --- | --- | --- | --- | --- | --- |
| **Pig farms** |  |  |  |  |  |
| All | 2575 | 187 (7.3%) | 0 (0.0%) | 34 (1.3%) | 4 (0.2%) |
| Feces | 2083 | 158 (7.6%) | 0 (0.0%) | 25 (1.2%) | 2 (0.1%) |
| Soils | 127 | 12 (9.4%) | 0 (0.0%) | 3 (2.4%) | 2 (1.6%) |
| Dusts | 170 | 5 (2.9%) | 0 (0.0%) | 1 (0.6%) | 0 (0.0%) |
| Sewages | 136 | 9 (6.6%) | 0 (0.0%) | 4 (2.9%) | 0 (0.0%) |
| Vegetables | 59 | 3 (5.1%) | 0 (0.0%) | 1 (1.7%) | 0 (0.0%) |
| **Migratory birds** |  |  |  |  |  |
| All | 972 | 0 (0.0%) | 5 (0.5%) | 2 (0.2%) | 0 (0.0%) |
| Feces | 863 | 0 (0.0%) | 5 (0.6%) | 2 (0.2%) | 0 (0.0%) |
| Water | 54 | 0 (0.0%) | 0 (0.0%) | 0 (0.0%) | 0 (0.0%) |
| Soils | 55 | 0 (0.0%) | 0 (0.0%) | 0 (0.0%) | 0 (0.0%) |
| **Human samples or isolates** |  |  |  |  |  |
| All | 701 | 1 (0.1%) | 0 (0.0%) | 0 (0.0%) | 0 (0.0%) |
| Urine of physical examination people | 175 | 1 (0.6%) | 0 (0.0%) | 0 (0.0%) | 0 (0.0%) |
| Nasal swabs of inpatients | 64 | 0 (0.0%) | 0 (0.0%) | 0 (0.0%) | 0 (0.0%) |
| Rectal swabs of inpatients | 60 | 0 (0.0%) | 0 (0.0%) | 0 (0.0%) | 0 (0.0%) |
| *A. baumannii* isolates from inpatients | 402 | 0 (0.0%) | 0 (0.0%) | 0 (0.0%) | 0 (0.0%) |

**Table S3 MICs of tetracyclines for the studied strains.**

| Strains | Sources | MICs (µg/mL)^a^ | | | | | |
| --- | --- | --- | --- | --- | --- | --- | --- |
|  |  | TC | DOX | MIN | TGC | ERA | OMA |
| *E. coli* JM109+pBAD24 | Empty vector | 2 | 0.5 | 0.25 | 0.13 | 0.008 | 0.13 |
| *E. coli* JM109+pBAD24-*tet*(X3) | *tet*(X3) clone | 256 | 64 | 32 | 16 | 4 | 16 |
| *E. coli* JM109+pBAD24-*tet*(X4) | *tet*(X4) clone | 256 | 64 | 32 | 16 | 4 | 16 |
| *E. coli* JM109+pBAD24-*tet*(X5) | *tet*(X5) clone | 128 | 32 | 16 | 8 | 4 | 8 |
| *E. coli* JM109+pBAD24-*tet*(X5.2) | *tet*(X5.2) clone | 128 | 32 | 16 | 8 | 4 | 16 |
| *E. coli* JM109+pBAD24-*tet*(X5.3) | *tet*(X5.3) clone | 128 | 32 | 16 | 8 | 4 | 16 |
| *E. coli* JM109+pBAD24-*tet*(X3)/*tet*(X5.2) | *tet*(X3)/*tet*(X5.2) clone | 256 | 64 | 32 | 16 | 4 | 16 |
| *E. coli* JM109+pBAD24-*tet*(X3)/*tet*(X5.3) | *tet*(X3)/*tet*(X5.3) clone | 256 | 64 | 32 | 16 | 4 | 16 |
| *E. coli* JM109+pBAD24-*fmo* | Ancestor clone | 2 | 0.5 | 0.25 | 0.13 | 0.008 | 0.13 |
| *A. baylyi* ADP1 | Laboratory strain | 0.5 | ≤0.06 | 0.13 | 0.06 | ≤0.008 | ≤0.008 |
| *Acinetobacter* spp.10FS3-1 [*tet*(X3)] | Pig | 128 | 32 | 16 | 32 | 8 | 16 |
| *A. baylyi* ADP1+p10FS3-1-3 | Transconjugant | 128 | 32 | 8 | 8 | 4 | 8 |
| *A. piscicola* YH12207 [*tet*(X3)/*tet*(X5.3)] | Pig | 64 | 16 | 8 | 8 | 4 | 16 |
| *A. baylyi* ADP1+pYH12207-2 | Transconjugant | 64 | 32 | 8 | 8 | 4 | 8 |

^a^TC, tetracycline; DOX, doxycycline; MIN, minocycline; TGC, tigecycline; ERA, eravacycline; OMA, omadacycline.

**References**

[1] Robert X, Gouet P. Deciphering key features in protein structures with the new ENDscript server. Nucleic Acids Res. 2014;42:W320-4.

[2] Xu YC, Liu LZ, Sun J, Feng YJ. Limited distribution and mechanism of the TetX4 tetracycline resistance enzyme. Sci Bull. 2019;64:1478-81.

[3] Waterhouse A, Bertoni M, Bienert S, Studer G, Tauriello G, Gumienny R, et al. SWISS-MODEL: homology modelling of protein structures and complexes. Nucleic Acids Res. 2018;46:W296-W303.

[4] Sun J, Chen C, Cui CY, Zhang Y, Liu X, Cui ZH, et al. Plasmid-encoded *tet*(X) genes that confer high-level tigecycline resistance in *Escherichia coli*. Nat Microbiol. 2019;4:1457-64.

[5] Li Y, Shen Z, Ding S, Wang S. A TaqMan-based multiplex real-time PCR assay for the rapid detection of tigecycline resistance genes from bacteria, faeces and environmental samples. BMC Microbiol. 2020;20:174.

[6] Lian X, Wang X, Liu X, Xia J, Fang L, Sun J, et al. *oqxAB*-positive IncHI2 plasmid pHXY0908 increase *Salmonella enterica* serotype Typhimurium strains tolerance to ciprofloxacin. Front Cell Infect Microbiol. 2019;9:242.
